# Validation of a model to investigate the effects of modifying cardiovascular disease (CVD) risk factors on the burden of CVD: the rotterdam ischemic heart disease and stroke computer simulation (RISC) model

**DOI:** 10.1186/1741-7015-10-158

**Published:** 2012-12-06

**Authors:** Bob JH van Kempen, Bart S Ferket, Albert Hofman, Ewout W Steyerberg, Ersen B Colkesen, S Matthijs Boekholdt, Nicholas J Wareham, Kay-Tee Khaw, MG Myriam Hunink

**Affiliations:** 1 Department of Epidemiology, Erasmus MC Rotterdam, dr Molewaterplein 50, Rotterdam, 3015 GE, the Netherlands; 2 Department of Radiology, Erasmus MC Rotterdam, dr Molewaterplein 50, Rotterdam, 3015 GE, the Netherlands; 3 Department of Public Health, Erasmus MC Rotterdam, dr Molewaterplein 50, Rotterdam, 3015 GE, the Netherlands; 4 Department of Health Policy and Management, Harvard School of Public Health, 677 Huntington Avenue, Boston, MA 02115, USA; 5 Department of Cardiology, Amsterdam Medical Center, Meibergdreef 9, Amsterdam, 1150 AZ, the Netherlands; 6 Department of Cardiology, Antonius Hospital, Koekoekslaan 1, Nieuwegein, 3435 CM, the Netherlands; 7 Medical Research Council Epidemiology Unit, Hills Road, Cambridge, CB2 0QQ, UK; 8 Department of Public Health and Primary Care, University of Cambridge, Robinson Way, Cambridge, CB2 0SR, UK

**Keywords:** Cardiovascular disease prevention, Simulation modeling, Model validation

## Abstract

**Background:**

We developed a Monte Carlo Markov model designed to investigate the effects of modifying cardiovascular disease (CVD) risk factors on the burden of CVD. Internal, predictive, and external validity of the model have not yet been established.

**Methods:**

The Rotterdam Ischemic Heart Disease and Stroke Computer Simulation (RISC) model was developed using data covering 5 years of follow-up from the Rotterdam Study. To prove 1) internal and 2) predictive validity, the incidences of coronary heart disease (CHD), stroke, CVD death, and non-CVD death simulated by the model over a 13-year period were compared with those recorded for 3,478 participants in the Rotterdam Study with at least 13 years of follow-up. 3) External validity was verified using 10 years of follow-up data from the European Prospective Investigation of Cancer (EPIC)-Norfolk study of 25,492 participants, for whom CVD and non-CVD mortality was compared.

**Results:**

At year 5, the observed incidences (with simulated incidences in brackets) of CHD, stroke, and CVD and non-CVD mortality for the 3,478 Rotterdam Study participants were 5.30% (4.68%), 3.60% (3.23%), 4.70% (4.80%), and 7.50% (7.96%), respectively. At year 13, these percentages were 10.60% (10.91%), 9.90% (9.13%), 14.20% (15.12%), and 24.30% (23.42%). After recalibrating the model for the EPIC-Norfolk population, the 10-year observed (simulated) incidences of CVD and non-CVD mortality were 3.70% (4.95%) and 6.50% (6.29%). All observed incidences fell well within the 95% credibility intervals of the simulated incidences.

**Conclusions:**

We have confirmed the internal, predictive, and external validity of the RISC model. These findings provide a basis for analyzing the effects of modifying cardiovascular disease risk factors on the burden of CVD with the RISC model.

## Background

Decision models are being increasingly used to guide decisions on medical interventions in healthcare [1-3]. Both for healthcare policy-makers who have to make decisions for specific populations and weigh both benefits and costs, and for a general practitioner facing a medical decision for a particular patient, decision models can provide valuable information to aid the decision at hand. Empirical and trial-based studies on (cost-)effectiveness of medical interventions often evaluate a limited number of strategies, and typically cover a limited period of follow-up. Decision modeling can overcome these limitations by synthesizing the available information and extrapolating short-term study results, providing policy-makers with information on expected long-term outcomes and accompanying uncertainties [4]. However, because decision models are based on a necessarily simplified representation of the underlying disease and the intervention being studied, the validity of the model is not automatically guaranteed. Earlier research has shown that importance of model validation before the results of a simulation study can be used for medical decisions [5-8].

Three types of validity have been described. With internal validation, the output of the model is compared with the data that was used to build the model [9,10]. Although model output and data are inherently dependent on each other with this type of validation, internal validity is a necessary condition, and provides an indication of how well the model output represents the data. Whereas the follow-up period in observational studies and clinical trials is necessarily limited, medical decisions often require long-term outcomes. A common approach is to extrapolate the results of a simulation model beyond the period on which it was originally based. The validity of a model with regard to its accuracy to simulate results beyond the original timeframe is called 'predictive' or 'prospective' validity [11,12], and constitutes the second form of validity. In evaluating predictive validity, the model output is compared with data from the new follow-up period, which has become available after the model was developed. The extent to which the results of a model can be applied to other populations different from the original one is the third form of validity, external validity [9,10]. Because potential differences between populations affect many of the parameters used in a model, external validity is a more rigorous test of model validity than the other two validity measurements.

The objective of this study was to assess the internal, predictive, and external validity of the Rotterdam Ischemic Heart Disease and Stroke Computer Simulation (RISC) model [13]. The RISC model was designed to investigate the effects of modifying cardiovascular disease (CVD) risk factors on the CVD burden in a general population. The model is based on data from the Rotterdam Study, a cohort follow-up study of 7,983 adults aged 55 years and older. Validation of the RISC model is required before the results produced by the model can be used for decision-making.

## Methods

### The model

**Figure 1:**
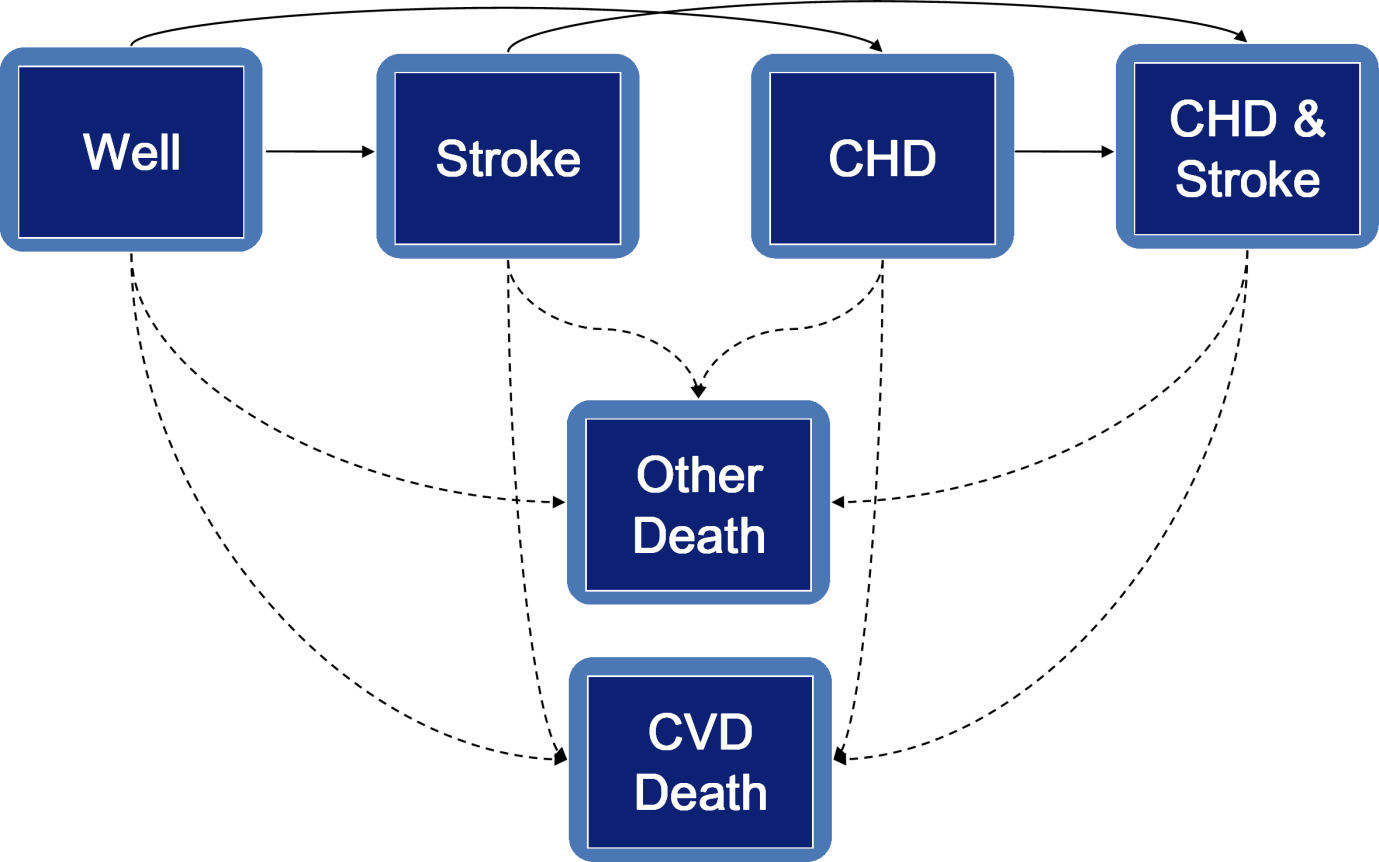
**Schematic presentation of the Rotterdam Ischemic Heart Disease and Stroke Computer Simulation (RISC) model**. CHD, coronary heart disease; CVD, cardiovascular disease. Arrows indicate transitions between the health states.

The RISC model is a Monte Carlo state-transition model (schematically presented in Figure 1) with six states: 1) the CVD death state, 2) the non-CVD death state, 3) the coronary heart disease (CHD) state, 4) the stroke state, 5) the CHD and stroke state, and 6) the well state (being alive without CHD or stroke). The model simulates incident CVD events in individuals with and without previous CVD based on risk-actor-dependent transition probabilities, using Cox regression equations.

Individual risk-factor profiles are modeled and tracked over time. Incident CVD events are counted using tracker variables during the period of simulation. CHD is defined as: acute myocardial infarction (*International Classification of Diseases*, 10th edition (ICD-10) code I21), percutaneous transluminal coronary angioplasty (PTCA) and coronary artery bypass graft (CABG). Stroke is limited to non-hemorrhagic and unspecified strokes (ICD-10 codes I63, I64). Cardiovascular death is defined as mortality due to hypertensive diseases (ICD-10 codes I10 to 15), ischemic heart disease (ICD-10 codes I20 to I25), sudden cardiac death (ICD-10 codes I46, I49), congestive heart failure (ICD-10 code I50), cerebrovascular disease (ICD-10 codes 160 to 167), other arterial disease (ICD-10 codes I70 to I79), or sudden death (ICD-10 code R96). Non-cardiovascular death is defined as mortality due to all other causes (all other ICD-10 codes). The model was built using TreeAge software (version Data Professional release 2009; TreeAge Software, Inc., Williamstown, USA). Detailed information about the model has been given in an earlier publication [13] (see also Additional file 1).

### Ethics approval

In the RISC model, the risk-factor profiles and transition probability functions were based on data from the Rotterdam Study population. The Rotterdam Study was originally approved by the institutional review board of the Erasmus Medical Center and by the review board of The Netherlands Ministry of Health, Welfare and Sports [14].

### Data sources

This population consisted of 7,983 respondents from a random sample of adults aged 55 years and older, who were recruited between 1990 and 1993 and were residing in Ommoord, the Netherlands. Of these 7,983 respondents, 6,871 both visited the research center and signed an informed consent document. These individuals were followed up from 1990 to 2000; the follow-up consisted of three physical examinations with interviews, and the surveillance of hospital admissions, death registries, and other available medical sources ensured accurate follow-up of death and clinical manifestations of CVD.

In 3,501 of the participants, all important characteristics for prediction of CVD were known, and the RISC model is based on 5-year follow-up data from these 3,501 individuals. The risk factors considered for the transition probability functions were age, sex, smoking status, systolic and diastolic blood pressure, body mass index, waist-to-hip ratio, ankle-brachial index; levels of plasma glucose, plasma total cholesterol, high-density lipoprotein (HDL) cholesterol, and plasma creatinine; family history of CVD, presence of hypertension (blood pressure over 160/90 or use of anti-hypertensive medication) or diabetes mellitus; manifestations of intermittent claudication, angina pectoris, atrial fibrillation or transient ischemic attacks; and prevalent CVD. Details about the assessment of these risk indicators have been described in earlier publications [15]. The Cox regression equations that described the state-transition probabilities were centered around the mean of the risk factors of these 3,501 participants. This enabled the analysis of populations other than the original one, by substituting the centered cumulative baseline hazard and the average values of the risk factors by the values from the other population(s).

### Simulation of parameter uncertainty

The RISC model allows for the evaluation of parameter uncertainty [16]. The majority of the parameter uncertainty in the model stems from the β-coefficients underlying the transition probability functions, and these β-coefficients are potentially dependent on each other. To model the uncertainty of the coefficients, 100 bootstrap samples of the study population were drawn. All the transition probability functions were fitted for every bootstrap sample, resulting in 100 sets of linked transition probability functions, which allowed for the dependency between them. The transition probabilities were based on Cox regression equations, and parameter uncertainty around the baseline hazards of the CVD events, CVD death, and non-CVD death was also included.

### Simulation of heterogeneity

The RISC model was designed to simulate individuals who each had a unique risk-factor profile for CVD [17]. Model outcomes are expected to be different for individuals with high-risk profiles (older age, male, high blood pressure, high lipid levels, diabetes mellitus) than for those with more favorable profiles. To allow for differences in outcomes resulting from individual differences in risk-factor profiles (that is, heterogeneity), we used the RISC model to simulate different individuals one at a time.

### Simulation of the history for each individual

The risk factors used in the RISC model reveal trends over time. As an example, total cholesterol levels were found decline with age in the Rotterdam Study. To take these trends in risk factors over time into account, each risk-factor profile for a particular individual was updated every 5 years during their simulated life in the model, based on the trends seen during the first 5 years in the Rotterdam Study. Therefore, the development of the risk factors needed to be tracked over time.

Events occurring during an individual's simulated life could influence the occurrence of other events. As an example, a CHD event increases the risk of dying in subsequent years. All cardiovascular events in the RISC model were therefore tracked and linked to the transition probabilities. The inclusion of variables used to track CVD events and changes in risk factors over time for each individual required the simulation of each individual multiple times to account for stochastic uncertainty [17].

### Internal and predictive validation

From our cohort of 3,501 individuals from the Rotterdam Study on which the RISC model was based, we selected 3,478 who had at least 13 years of follow-up as of 1 January 2007. The remaining subjects were lost to follow-up because they had moved out of the area or had discontinued their participation. We calculated the cumulative incidences for total mortality, CVD mortality, non-CVD mortality, CHD, and stroke as defined previously for the 13-year period of follow-up (beginning of year 1 until end of year 13). We then compared this with the simulated cumulative incidences of the same events during the 1^st ^year until the end of the 13^th ^year by the RISC model. We furthermore stratified the analyses for the internal and predictive validity for CVD mortality by tertiles of age for the 3,501 participants, and for men and women separately. We choose CVD mortality because it is one of the most important clinical outcomes, and there would be enough events for it in each stratum to obtain stable results.

### External validation

For the external validation, we used data from the EPIC-Norfolk study [18], which is a prospective population study of 25,663 men and women aged 45 to 79 years old residing in Norfolk, UK. This study had been approved by the Norwich District Health Authority ethics committee, and all participants gave signed informed consent [18]. Participants were originally recruited from age and gender registers of general practices in Norfolk as part of the 10-country collaborative EPIC study designed to investigate dietary and other determinants of cancer. Additionally, characteristics including anthropometry, blood pressure, and lipid levels were obtained for the assessment of determinants of other diseases. For the baseline survey from 1993 to 1997, participants completed a detailed health and lifestyle questionnaire and attended a clinic visit. All participants were followed up and mortality, linked to the UK Office of National Statistics, was recorded. Participants admitted to hospital were identified by their unique National Health Service number by data linkage with the East Norfolk Health Authority (ENCORE) database, which identifies all hospital contacts throughout England and Wales for Norfolk residents.

The EPIC data did not contain all variables used in the RISC model. In particular, the following information was not readily available: ankle-brachial index, serum glucose levels, and a history at baseline of angina pectoris, atrial fibrillation, intermittent claudication, or transient ischemic attack. Consequently, we imputed the missing data in the EPIC dataset based on the multiple variables that were available [19]. All major risk factors such as age, sex, cholesterol levels, and blood pressure were available and did not need to be imputed.

We used EPIC-Norfolk mortality data from 1993 until 31 March 2008. From the 25,663 participants, we selected 25,492 who had a follow-up of at least 10 years. For the external validation, we calculated the cumulative incidence of CVD and non-CVD mortality in the EPIC dataset. We compared this with the simulated cumulative incidences of the same events after year 1 until year 10 by the RISC model, using the 25,492 EPIC profiles as input.

We did not calculate or simulate CHD and stroke events in the external validation, because the EPIC study did not document CABG and PCI events and furthermore, non-fatal events were only recorded if the patient was hospitalized. In the Rotterdam Study, both CABG and PCI were counted as CHD events, and all CHD and stroke events were recorded whether or not the patient was hospitalized, making the definition of CHD and stroke inherently different between the two cohorts [20,21].

### Statistical analysis

Important baseline characteristics for the baseline 3,478 Rotterdam Study participants and 25,492 EPIC participants were calculated and tabulated to evaluate their differences.

To take into account parameter uncertainty, the heterogeneity of the participants, and the stochastic uncertainty, we performed a three-level simulation [16,17]. We calculated the mean and distribution around the mean of the cumulative incidences by drawing from 100 second-order sets of linked β-coefficients from the state-transition probabilities and values for the baseline hazards of the events (outer simulation loop for parameter uncertainty). For each set of linked β-coefficients and baseline hazards, we consecutively simulated 2,000 randomly drawn risk-factor profiles from the 3,478 Rotterdam profiles for the internal and predictive validation, and 2,000 from the 25,492 EPIC profiles for the external validation (middle simulation loop for heterogeneity). For each profile, 200 random walks were simulated, needed for the tracking of the individual cardiovascular histories (microsimulation, inner simulation loop for stochastic uncertainty). This implies 100 × 2,000 × 200 runs per analysis. We did not model any particular intervention or treatment in this study; only the observed history (current practice) was simulated for purposes of validation. For the stratified analyses we aggregated on the individual level (n = 3,501 × 200 × 100 runs per analysis).

For the internal and predictive validation, we determined the average simulated cumulative incidences of CVD death, non-CVD death, CHD, and stroke for the 13-year period. For the external validation, we determined the average simulated cumulative incidences of CVD death and non-CVD death for year 1 until year 13. Because the Rotterdam Study and EPIC-Norfolk population are potentially different with respect to the distribution of risk factors and incidence of CVD, we subsequently recalibrated the RISC model by substituting the centered cumulative baseline hazards and mean values of the risk factors from the original model based on the Rotterdam data with the corresponding ones from the EPIC-Norfolk cohort [22]. We then ran again 2,000 randomly drawn participants from the 25,492 EPIC participants.

For all cumulative incidences, we calculated the 2.5% and 97.5% percentiles of the variation around the average incidences (credibility intervals) from the RISC simulations, to quantify the influence of parameter uncertainty. We compared the observed with the simulated incidences for all events.

## Results

Compared with the Rotterdam Study, the the EPIC-Norfolk study participants were 10 years younger on average, and there were more men in the EPIC-Norfolk study (Table 1). On average, EPIC participants had lower total cholesterol levels and higher HDL levels (Table 1). The number of Rotterdam Study participants with a history of CVD at baseline exceeded that of the EPIC participants.

**Table 1:** Baseline characteristics of the risk factors used in the Rotterdam Ischemic Heart Disease and Stroke Computer Simulation (RISC) model for the 3,478 Rotterdam study participants and 25,492 European Prospective Investigation of Cancer (EPIC)-Norfolk study participants

Variable	RISC (n = 3,478)	EPIC (n = 25,492)
Age	69.0 (62 to 75)	59.2 (51 to 67)

Male subjects, %	39%	45%

Smoker		

Never	34.5%	46.0%

Former	41.9%	42.3%

Current	23.6%	11.7%

BMI	26.3 (23.8 to 28.5)	26.3 (23.7 to 28.4)

WHR	0.91 (0.84 to 0.97)	0.86 (0.78 to 0.93)

Systolic BP	140.0 (124 to 155)	135.5 (122.5 to 146.5)

Diastolic BP	74.1 (66 to 82)	82.5 (74.5 to 89.5)

Hypertension	36.4%	29.9%

Total cholesterol	6.67 (5.8 to 7.4)	6.19 (5.4 to 6.9)

HDL cholesterol	1.34 (1.1 to 1.5)	1.41 (1.1 to 1.6)

Glucose^b^	6.93 (5.5 to 7.5)	6.67 (5.5 to 7.3)

Creatinine	82.5 (72 to 91)	86.7 (76 to 97)

Diabetes mellitus	10.7%	12.2%

Angina pectoris^b^	10.4%	9.2%

Atrial fibrillation^b^	2.5%	2.9%

Intermittent claudication^b^	2.1%	1.5%

TIA^b^	5.1%	4.8%

CVD	17.8%	4.3%

Family history of MI	16.3%	18.4%

Family history of CVD	23.0%	23.3%

Abbreviations: BMI, body mass index; BP, blood pressure; CVD, cardiovascular disease; MI, myocardial infarction; TIA, transient ischemic attack; WHR, waist-to-hip ratio.

^a^Average values and inter-quartile ranges (brackets) are given for continuous variables, while categorical variables are given as percentages.

^b^Indicates imputed risk factors for the EPIC-Norfolk dataset.

### Internal and predictive validation

**Figure 2:**
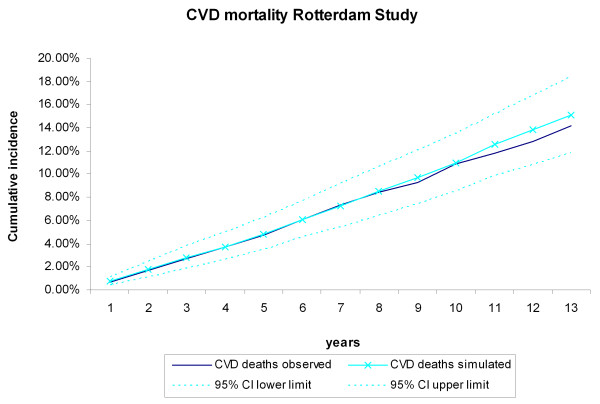
**Cardiovascular disease (CVD) mortality during 13 years of follow-up**. The first 5 years refer to the internal validation, the remaining years to the predictive validation. Simulated versus observed values for the Rotterdam Study data.

**Figure 3:**
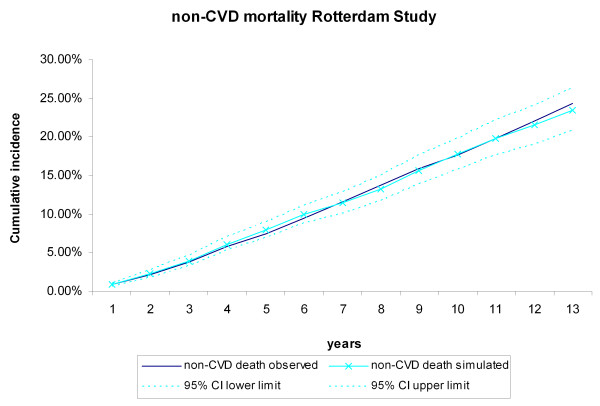
**Non-cardiovascular disease (CVD) mortality during 13 years of follow-up**. The first 5 years refer to the internal validation, the remaining years to the predictive validation. Simulated versus observed values for the Rotterdam Study data.

**Figure 4:**
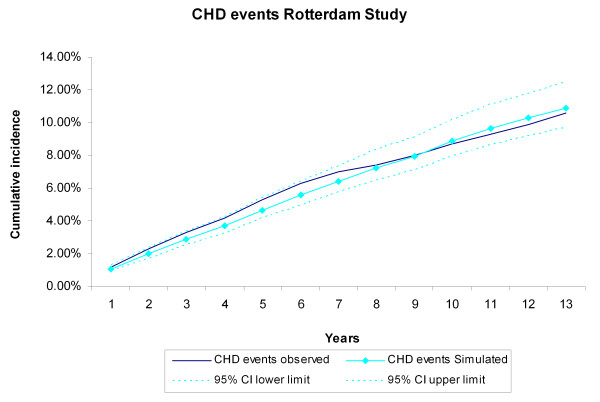
**Coronary heart disease (CHD) events during 13 years of follow-up**. The first 5 years refer to the internal validation, the remaining years to the predictive validation. Simulated versus observed values for the Rotterdam Study data.

**Figure 5:**
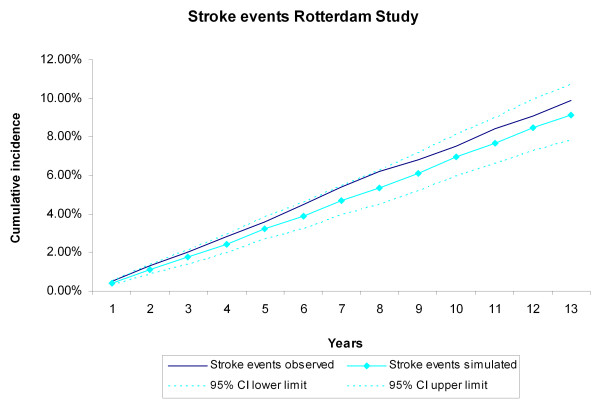
**Stroke events during 13 years of follow-up**. The first 5 years refer to the internal validation, the remaining years to the predictive validation. Simulated versus observed values for the Rotterdam Study data.

During the 13 years of follow-up, 367 CHD events, 343 stroke events, 494 CVD deaths, and 846 non-CVD deaths occurred in the 3,478 Rotterdam Study participants, The cumulative incidences of CVD and non-CVD mortality during the13 years of follow-up for the Rotterdam Study participants were compared with the incidences generated by the RISC model (Figure 2, Figure 3). The observed values, both during the first 5 years (internal validation) and for the extrapolated period (predictive validation), were consistent with the simulated ones. The cumulative incidences of CHD and stroke events during the 13-year follow-up were compared with the incidences generated by the RISC model (Figure 4, Figure 5). The observed values were again consistent with the simulated events. For the cumulative incidences of CVD mortality, stratified by tertiles of age, for men and women respectively, the observed values were also consistent with the simulated values (see Additional file 1, Figure S2, Figure S3).

### External validation and recalibration

**Figure 6:**
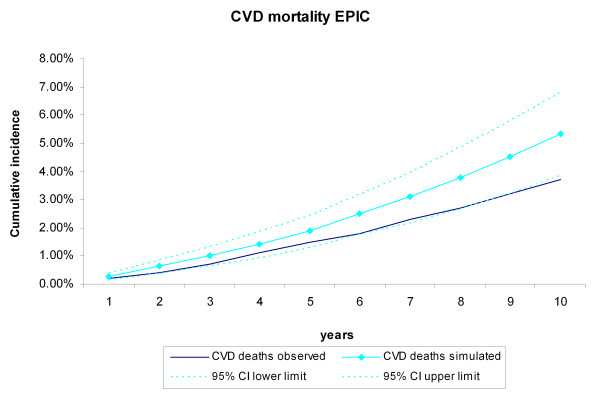
**Cardiovascular disease (CVD) mortality during 10 years of follow-up**. Simulated versus observed values for the European Prospective Investigation of Cancer (EPIC)-Norfolk data.

**Figure 7:**
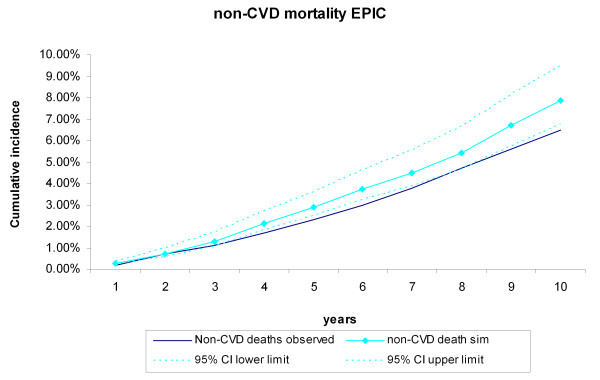
**Non-cardiovascular disease (CVD) mortality during 10 years of follow-up**. Simulated versus observed values for the European Prospective Investigation of Cancer (EPIC)-Norfolk data.

**Figure 8:**
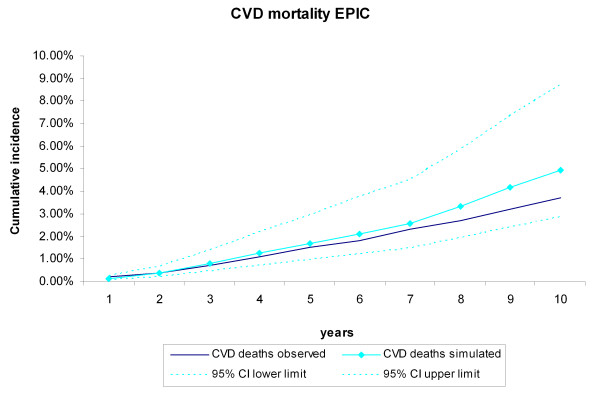
**Cardiovascular disease (CVD) mortality during 10 years of follow-up in the recalibrated model**. Simulated versus observed values for the European Prospective Investigation of Cancer (EPIC)-Norfolk data.

**Figure 9:**
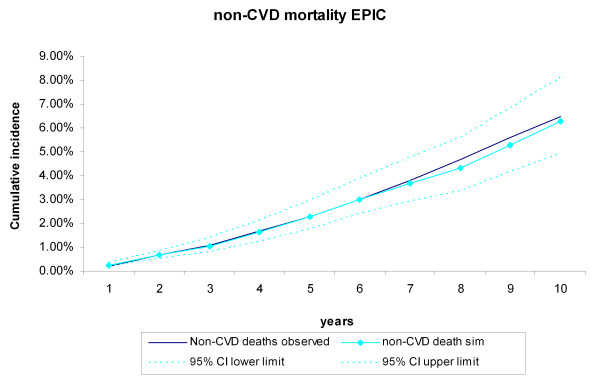
**Non-cardiovascular disease (CVD) mortality during 10 years of follow-up in the recalibrated model**. Simulated versus observed values for the European Prospective Investigation of Cancer (EPIC)-Norfolk data.

During the 10-year follow-up of the 25,492 EPIC-Norfolk participants, 943 CVD deaths and 1,661 non-CVD deaths occurred. The cumulative incidence of CVD and non-CVD mortality during the 10-year follow-up of the 25,492 EPIC participants were compared with the incidences generated by the RISC model, using the EPIC-Norfolk profiles as input (Figure 6, Figure 7). The observed values were within the 95% credibility intervals of the simulated values, but the RISC model overestimated the incidences for all years, for both CVD and non-CVD mortality. We then estimated the cumulative incidences of CVD and non-CVD mortality, after substituting the centered cumulative baseline hazards and average values of the risk factors with those based on the EPIC data, which recalibrated the model (Figure 8, Figure 9). After this recalibration, the observed CVD and non-CVD mortality incidences matched the simulated incidences from the RISC model.

## Discussion

In this study, we evaluated the internal, predictive, and external validity of the RISC model. The simulated cumulative incidence of CVD and non-CVD deaths, CHD events, and strokes adequately represented the data during the original follow-up period of 5 years on which the RISC model was based. Extrapolation of the simulated results beyond this period proved to be valid for 13 years of follow-up, the maximum length that we analyzed in this paper. Although the results of the RISC model overestimated the CVD and non-CVD mortality compared with the observed 10-year incidences in the EPIC-Norfolk population, recalibrating the model with the cumulative baseline hazards and mean values of the risk factors substantially improved performance.

Other decision models used to evaluate preventive and treatment strategies for CVD have been well established. A recent review by Unal *et al. *identified forty-two such models, of which six major ones have been described in detail [23]. Although some of the forty-two models reported assessment of validity, most did not. Of the six major models, three have not been validated [24-26], two models had information on internal validity reported [27,28], and an external validation had been performed fo two models [29,30].

In the present study, the predictive validity of the RISC model was tested against follow-up data for more than twice the length of the period on which the model was originally based. The fact that the observed and simulated incidences matched closely even when extrapolated beyond the original data makes it plausible to expect projections beyond 13 years to be valid as well. The trends in risk factors over time and their effects on the incidence of events, which are jointly modeled in the RISC model, seem to provide a valid basis to extrapolate results, without the need to recalibrate the model for the Rotterdam Study population. We furthermore showed the robustness of the internal and predictive validity by providing results for the stratified analyses by tertiles of age and sex. As for the external validation, the EPIC-Norfolk population was on average younger and healthier than the Rotterdam Study population. It was to be expected that an unadjusted model, using the baseline hazards and mean of the risk factors from the Rotterdam Study, would overestimate the observed incidences in the EPIC-Norfolk study. In the recalibrated model, we updated only the baseline cumulative hazards of the events and the mean values of the risk factors, a method very commonly used when applying models to other populations than that for which the model was originally developed in [22,31]. This result suggests that the relative strengths of the associations of the risk factors with the incidence of the events in the RISC model are the same for both the EPIC-Norfolk population and Rotterdam Study. The resulting external validity of the RISC model after this adjustment strongly supports this assumption.

Our analysis does have some limitations. The RISC model was designed to investigate the effects of modifying cardiovascular risk factors on the burden of CVD in the middle-aged and older general population. We validated the model in the EPIC-Norfolk data, which included people aged from 45 years upwards. Although most current guidelines on the primary prevention of CVD mostly start at the age of 45 years and older, some do (or in the future potentially will), suggest that CVD prevention should begin at an earlier age Whether the RISC model also performs well in a younger population remains to be determined. The RISC model is intended to be used for projections during the remaining lifetime of an individual. The model proved to be valid for projections during 13 years of follow-up, and for most older people this is sufficiently long to cover their remaining lifespan. For younger people, this is less likely, and model extrapolation beyond this period therefore has to be made, which currently has not been validated. Because the Rotterdam Study is ongoing, and longer follow-up data are being collected, we will be able to test whether this additional extrapolation is valid as well.

A number of risk factors used for the RISC model were not documented in the EPIC-Norfolk study. To make the EPIC-Norfolk dataset suitable for the RISC model, we imputed missing data based on the correlations between the missing risk factors and the documented variables. These correlations stemmed from the Rotterdam Study data, thereby introducing dependency between the (imputed) EPIC-Norfolk data and the RISC model. However, the major traditional risk factors such as age, sex, cholesterol level, and blood pressure were available in EPIC. The prevalence of a number of missing risk factors such as atrial fibrillation and intermittent claudication were low in the Rotterdam Study data on which the RISC model was developed, and the incremental value beyond the traditional risk factors of the other variables, such as the ankle-brachial index, has been found to be limited [32]. It is therefore less likely that the imputation influenced the external validity in favor of concordance. Although the EPIC-Norfolk dataset contains information on (hospitalized) patients with MI, the RISC model simulates CHD as a combined endpoint, including CABG and PCI. This is consistent with most clinical trials using similar combined endpoints. The design of the RISC model therefore did not allow for direct comparison of simulated MIs as a sole endpoint. Although acute MI is the major component of CHD, both CABG and PCI interventions are inherently different from acute MIs, and we therefore did not externally validate CHD events in the EPIC dataset.

At the time of this paper, we did not have datasets other than EPIC-Norfolk at our disposal to perform additional external validation. The fact that the RISC model, after updating the model with the baseline hazards and mean values for the risk factors from EPIC, proved to be valid for the EPIC-Norfolk cohort, does not automatically imply that it will be valid in other populations as well. The EPIC-Norfolk cohort was younger on average, and included more men than the RISC cohort. However, the fact that the cohort was different with regard to these important risk factors, and yet RISC still provided valid results, does make a strong case that the model will be valid for other cohorts as well. We do intend to validate the model with other data as they become available. Both the Rotterdam Study and the EPIC-Norfolk study were population-based studies and included individuals regardless of pre-existing risk-factor profiles or disease status. Although risk-factor distributions of the study participants might in principle be different from the populations they intend to represent, it is very likely that the RISC model is valid for most western European populations in general after adjusting for baseline hazards. A simpler model with a reduced set of parameters, excluding the less common ones such as atrial fibrillation and ankle-brachial index, would possibly allow for a more rapid validation process in other populations. In an ongoing effort to optimize our model, we also intend to make efforts to simplify our current model.

We modeled and validated the cardiovascular histories of the participants of the Rotterdam Study and EPIC-Norfolk cohort as they were observed; that is, without any interventions. Although the results with regard to this validity seem promising, the RISC model will be used to evaluate interventions for the primary prevention of CVD. In that case, the validity of the model to evaluate an intervention depends not only on the observed CVD history, but also on the extent to which other structural assumptions are made, such as modeling the treatment effect of an intervention [33]. A more extensive framework of model validation proposed by Kopec *et al. *[34] also includes between-model comparisons, and comparisons of evidence from examining the consequences of model-based decisions. Between-model comparisons are specifically useful when analyzing certain interventions compared with the natural history of the disease, as we did in the current analysis. Being a simplifying abstraction of reality, a model will be valid with regard to some (but not necessarily all) mechanisms or relationships seen in real life. Assumptions made to assure that particular mechanisms are characterized can cause the model to be less valid with regard to other possible mechanisms. This makes the modeling of complex interrelationships more of an art than an exact science. For each particular decision problem, it is important to determine the assumptions to which each approach is sensitive, determine the appropriateness of these assumptions, and judge the relevance of the model sensitivity to them in the context of the decision problem and the forthcoming decisions that will result from it.

## Conclusions

This study shows that the RISC model accurately predicts mortality and CVD events during the period of 5 years on which it is based (internal validity) and during an extended follow-up period up for 13 years (predictive validity). In addition, after recalibration, it accurately predicts mortality in the EPIC-Norfolk cohort as well (external validity). These findings provide a basis to generalize results from the RISC model.

## Abbreviations

CABG: Coronary artery bypass graft; CHD: Coronary heart disease; CVD: Cardiovascular disease; EPIC: European Prospective Investigation of Cancer Study; ICD: *International Classification of Diseases*; PCI: Percutaneous coronary intervention; RISC: Rotterdam Ischemic Heart Disease and Stroke Computer Simulation.

## Competing interests

The authors declare that they have no competing interests.

## Authors' contributions

MH coordinated and, with BK and BF, designed the study. BK and BF developed the simulation model and analyzed the data. AH, MB, EC, NW, and KK provided the data used in the model. BK and BF, MH, and ES contributed to the interpretation of the data. BK and BF wrote the manuscript. All authors read and approved the final manuscript

## Pre-publication history

The pre-publication history for this paper can be accessed here:

http://www.biomedcentral.com/1741-7015/10/158/prepub

## Supplementary Material

Additional file 1 **Technical appendix**. Technical appendix with additional information on the Rotterdam Ischemic Heart Disease and Stroke Computer Simulation (RISC) model and analyses. Click here for file
