# Supplementary material for: Validation of a model to investigate the effects of modifying cardiovascular disease (CVD) risk factors on the burden of CVD: the rotterdam ischemic heart disease and stroke computer simulation (RISC) model
Source: BMC Med. 2012 Dec 6;10:158. doi: 10.1186/1741-7015-10-158 (PMC3566939; doi:10.1186/1741-7015-10-158)
Supplement: Additional file 1 — Technical appendix. Technical appendix with additional information on the Rotterdam Ischemic Heart Disease and Stroke Computer Simulation (RISC) model and analyses. [file 1741-7015-10-158-S1.DOC]

### Technical Appendix

In this Appendix, we provide additional information on the RISC model and analyses.

**RISC Model**

The RISC model is a Monte Carlo state-transition model (schematically presented in Figure S1) with six states: (1) the CVD death state, (2) the non-CVD death state, (3) the Coronary Heart Disease (CHD) state, (4) the Stroke state, (5) the CHD and Stroke state and (6) the Well state (being alive without coronary heart disease or stroke). The model simulates incident CVD events in individuals based on risk factor dependent transition probabilities, using Cox regression equations. Individual risk factor profiles were modeled and tracked over time. The model was built in TreeAge (version 2009, TreeAge Software, Inc., Williamstown, USA).

Probabilities for the transitions between the six health states were based on six multivariable Cox regression equations. The development of these equations was described in a previous article on the RISC model (1). The first equation estimated the cumulative hazard from the Well state to the CHD state and from the Stroke state to the CHD & Stroke state. The second equation estimated the cumulative hazard from the Well state to the Stroke state and from the CHD state to the CHD & Stroke state. In developing these models, censoring was performed for an incident stroke and CHD respectively. In both equations, previous CHD and/or stroke were included as a covariable. The third and fourth equations estimated the 6-months cardiovascular mortality rate (case-fatality) after a CHD and stroke event respectively. Six-month case-fatalities were used as proxies for the immediate fatality rates of these events. The probability of dying from a fourth CHD event and third stroke event were assumed to be 100%. The fifth and sixth Cox regression equations estimated the cumulative hazards of the remaining CVD mortality, which is caused by other causes than CHD or stroke (see Table S1 for equations). For extrapolation to a lifelong follow-up, follow-up time was divided into 5-year intervals and a cycle length of one year was chosen. The first 5 years, baseline values of covariables were used together with the one-year cumulative hazards from the Cox models for each cycle. For the remaining follow-up, the same baseline one-year cumulative hazards were used, but values of the covariables were updated every 5 years by using multiple linear regression for continuous variables and logistic regression for dichotomized variables. From the outcomes of the logistic regression equations regarding dichotomized variables, binomial distributions were created. Every 5 year period during follow-up, presence (1 or 0) of the dichotomized variables was derived from these distributions. Cumulative hazards of fatal and non-fatal events were separately weighted for their total cumulative hazards to ascertain that all probabilities for respectively cardiovascular and non-cardiovascular mortality vs. survival and stroke and CHD events vs. disease-free survival summed to one. For each transition, cumulative hazards were converted to probabilities by exponentiation. Occurrences of events and duration in each health state were stored using Monte Carlo tracker variables to allow for the calculation of incidences of the different events. These tracker variables are variables used to count the occurrences of a state transition representing an event in TreeAge.

**Table S1** Model Input Parameters

| **Cox Proportional Hazards Equations** | **Equations*** |
| --- | --- |
| CHD: | Hazard function = baseline cumulative hazard x EXP(β1*male+β2*age+β3*age*age+β4*diabetes*glucose+β5*TC+β6*HDL+β7*PP+β8*PP*male+β9*angina+β10*ABI+β11*ABI*ABI+β12*smoking+β13*famhistMI+β14*CVD+β15*creat-β16) |
| Stroke: | Hazard function = baseline cumulative hazard x EXP(β1*male+β2*age+β3*hypertension+β4*hypertension*age+β5*SBP+β6*smoking+β7*famhistMI+β8*TIA+β9*CVD+β10*CVD*male+β11*AF+β12*ABI-β13) |
| 6-months CHD event mortality: | Hazard function = baseline cumulative hazard x EXP(β1*age+β2*diabetes*glucose+β3*hypertension+β4*hypertension*age+β5*creat-β6) |
| 6-months Stroke event mortality: | Hazard function = baseline cumulative hazard x EXP(β1*age+β2*smoking+β3*famhistCVD+β4*ABI+β5*TC+β6*creat+β7*HDL+β8*ABI*ABI+β9*age*HDL-β10) |
| CVD mortality: | Hazard function = baseline cumulative hazard x EXP(β1*age+β2*male+β3*diabetes+β4*HDL+β5*hypertension+β6*hypertension*age+β7*smoking+β8*CVD+β9*ABI+β10*ABI*AΒI+β11*AF+β12*AF*male+β13*age*AF+β14*male*CVD+β15*CVD*TC*TC-β16) |
| Non-CVD mortality: | Hazard function = baseline cumulative hazard x EXP(β1*male+β2*age+β3*diabetes*glucose+β4*TC+β5*smoking+β6*smoking*age+β7*BMI+β8*BMI*age+β9*WHR+β10*WHR*age+β11*WHR*CVD+β12*famhistCVD+β13*famhistCVD*age+β14*ABI+β15*ABI*age+β16*CVD-β17) |

*Beta coefficients were drawn from a table comprising estimated beta coefficients from Cox regression equations developed in 100 bootstrapped datasets.

Abreviations: ABI, ankle-brachial index; AF, atrium fibrillation; BMI, body mass index; CHD, coronary heart disease; C.I.’s, confidence intervals; creat, creatinine; CVD, cardiovascular disease; HDL, famhistMI, family history of myocardial infarction; high-density lipoprotein; famhistCVD, family history of cardiovascular disease; PP, pulse pressure; SBP, systolic blood pressure; TC, total cholesterol; TIA, transient ischaemic attack; WHR, waist-to-hip ratio

**Figure S1**. Schematic presentation of RISC model.


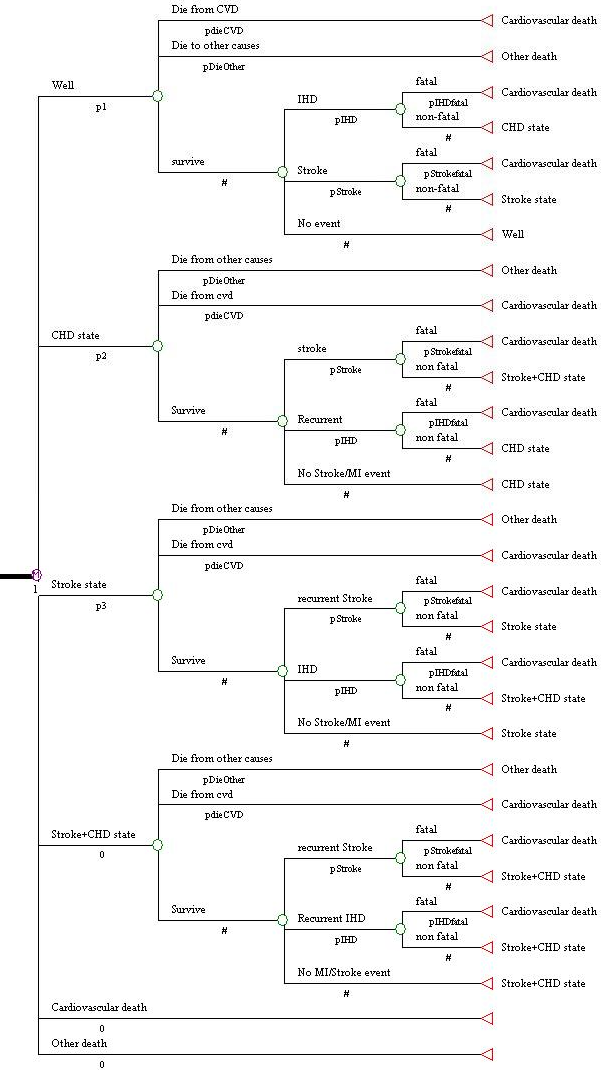


**Figure S2.** CVD mortality during 13 years of follow-up. Simulated vs observerd values per tertile of age in men for the Rotterdam Study data.

**Figure S3.** CVD mortality during 13 years of follow-up. Simulated vs observerd values per tertile of age in women for the Rotterdam Study data.

**References**

1. Nijhuis RL, Stijnen T, Peeters A, Witteman JC, Hofman A, Hunink MG. Apparent and internal validity of a Monte Carlo-Markov model for cardiovascular disease in a cohort follow-up study. Med Decis Making 2006;26:134-44.

2. Pletcher MJ, Lazar L, Bibbins-Domingo K, et al. Comparing impact and cost-effectiveness of primary prevention strategies for lipid-lowering. Ann Intern Med 2009;150:243-54.
